# *Ex Vivo* Liver Resection and Autotransplantation as Surgical Option for Zone II–III Leiomyosarcoma of IVC: A Case Report and Literature Review

**DOI:** 10.3389/fonc.2021.690617

**Published:** 2021-06-11

**Authors:** Tuerhongjiang Tuxun, Tao Li, Shadike Apaer, Yi-Biao He, Lei Bai, Shen-Sen Gu, Zhi-Peng Wang, Qiang Huo, Jiang Wang, Jin-Ming Zhao

**Affiliations:** ^1^ Department of Liver Transplantation & Liver Surgery, Center of Organ Transplantation, 1^st^ Affiliated Hospital of Xinjiang Medical University, Urumqi, China; ^2^ Department of Cardiac Surgery, 1^st^ Affiliated Hospital of Xinjiang Medical University, Urumqi, China; ^3^ Department of Anesthesiology, 1^st^ Affiliated Hospital of Xinjiang Medical University, Urumqi, China

**Keywords:** leiomyosarcoma, *ex vivo* liver resection and auto transplantation, survival, recurrence, surgery

## Abstract

We report the first documented case of leiomyosarcoma at zone II-III of inferior *vena cava* with thrombi in three hepatic veins undergoing *ex vivo* liver resection and autotransplantation (ELRA) and hepatic veins thrombectomy. A 33-year-old female patient presented with abdominal distention and lower extremities edema. Abdominal wall varicosis and shifting dullness were positive on physical examination. Her liver function was classified as Child-Pugh B and a solid tumor at retro-hepatic *vena cava* extending to right atrium with thrombi in three hepatic veins were confirmed. The diagnosis of leiomyosarcoma with Budd-Chiari syndrome was highly suspected with preoperative ultrasound, echocardiogram, CT scan, and three-dimensional reconstruction. A zone II-III leiomyosarcoma of IVC origin was confirmed at surgery and *ex vivo* liver resection and autotransplantation, and hepatic vein thrombectomy with atrial reconstruction were performed under cardiopulmonary bypass (CPB). Operative time, anhepatic time, and CPB time were 12 h, 128 min, and 84 min, respectively. The patients experienced post-operative liver dysfunction and was cured with conservative therapy. Hepatic recurrence two years after surgery was managed with radiofrequency. The patient was alive with liver metastasis three years after surgery. Despite being regarded as an extremely aggressive procedure, ELRA could be considered in the treatment of advanced leiomyosarcoma with Budd-Chiari syndrome and hepatic vein thrombi.

## Introduction

Leiomyosarcoma is a rare malignant tumor originating from the smooth muscle, with a small subset of vascular origin, especially from IVC. Due to its insidious grow and lack of early specific symptoms, it has usually developed to a late stage when diagnosed (1). Leiomyosarcomas of IVC are categorized by the anatomical location into three zones. Zone I refer to the infrarenal portion of the IVC, Zone II from the hepatic veins to the renal veins, and Zone III from the right atrium to the hepatic veins (2). The surgical management of zone I-III leiomyosarcomas represents a real clinical challenge, since its extensive involvement to major vasculatures requires multi-organ resection and complex vascular reconstruction (3). However, considering the questionable effectiveness of chemo- and radio-therapy in such patients, surgery is the only curative option (4). First described by Pichlmayr (5), *ex vivo* liver resection and autotransplantation (ELRA) was introduced as an alternative to allotransplantation for end-stage alveolar echinococcosis (6), cholangiocarcinoma and colorectal metastasis (7). ELRA provides technical opportunity for both malignant and benign lesions with hepato-caval region, three hepatic veins, and hilar involvement. Therefore, leiomyosarcomas affecting hepato-caval region could be theoretically benefited from ELRA. Despite sporadic report of seven cases, the role of ELRA in the management of leiomyosarcoma is still lacking. Herein, we present the first documented case of zone II-III leiomyosarcoma with thrombi in three hepatic veins and Budd-Chiari syndrome undergoing ELRA, thrombectomy under CPB, and we consider the relevant literature on the topic.

## Case Presentation

**Figure 1:**
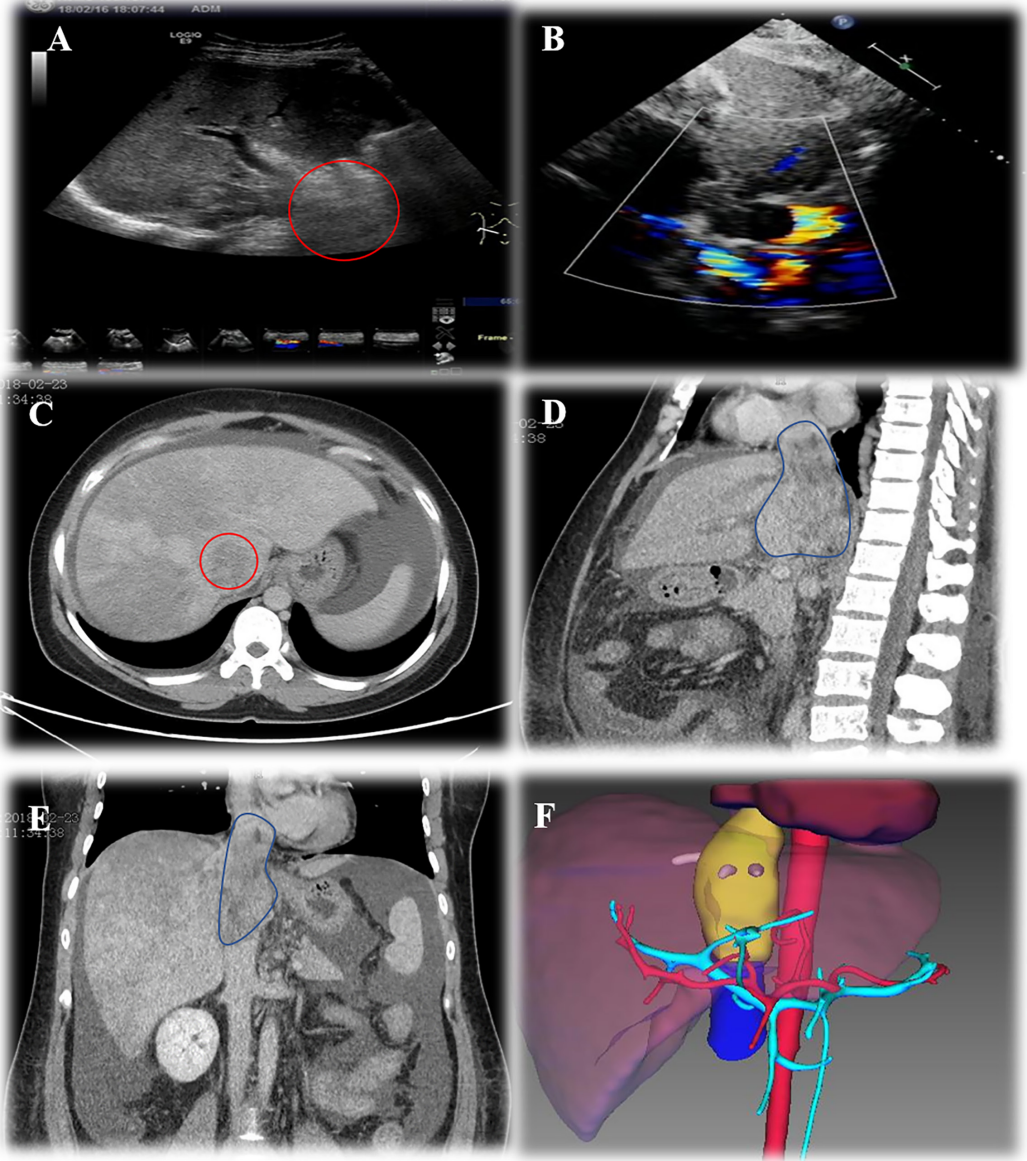
Preoperative assessment of Leiomyosarcoma. **(A)**, ultrasonography showed haeato-caval mass; **(B)**, echocardiogram showed lesion extending into right atrium; **(C)**, cross section CT scan showed lesion in IVC; **(D)**, sagittal section CT scan showed lesion extension and ascites; **(E)**, coronal section CT scan of IVC lesion; **(F)**, three-dimensional reconstruction showed lesion extension.

A 33-year-old female patient with progressive abdominal distention and leg edema for a month was referred to our center. Abdominal enlargement, abdominal wall varicosis, and shifting dullness were positive at physical examination. Laboratory results showed the following: hemoglobin, 108 g/L; albumin, 26 g/L, aspartate aminotransferase (AST), 145 IU/L; alanine aminotransferase (ALT), 165 IU/L; prothrombin time (PT), 16.8 s; PT activity, 46.46%; carbohydrate antigen-25 (CA125), 308 IU/ml. Ultrasonography showed ascites, hepatic congestion, and a solid tumor in retro-hepatic *vena cava* with thrombi in three hepatic veins. CT angiogram revealed a 10 cm × 6.5 cm × 4 cm occlusive mass at hepato-caval region with cephalad margin to right atrium. Echocardiogram revealed normal systolic and diastolic function with a 4.5 cm × 4 cm echo-dense mass in IVC extending into the mid-portion of the right atrium. Preoperative three-dimensional (3D) reconstruction showed the extension of tumor involvement (**Figure 1**).

A multi-disciplinary team consisting of members from Liver Transplantation, Surgical Oncology, Cardiothoracic surgery, Cardiothoracic Anesthesiology, and Hepatology reviewed patients’ general information and reports. Despite the compromised hepatic function of the patient, surgery was considered as the only curative option for solving the extending tumor and secondary Budd-Chiari syndrome. ELRA with bench thrombectomy was planned considering tumor involvement to three hepatic veins and right atrium as well as thrombi. Gore-Tex graft replacement of the IVC was considered if IVC-sparing tumor extraction was not possible.

**Figure 2:**
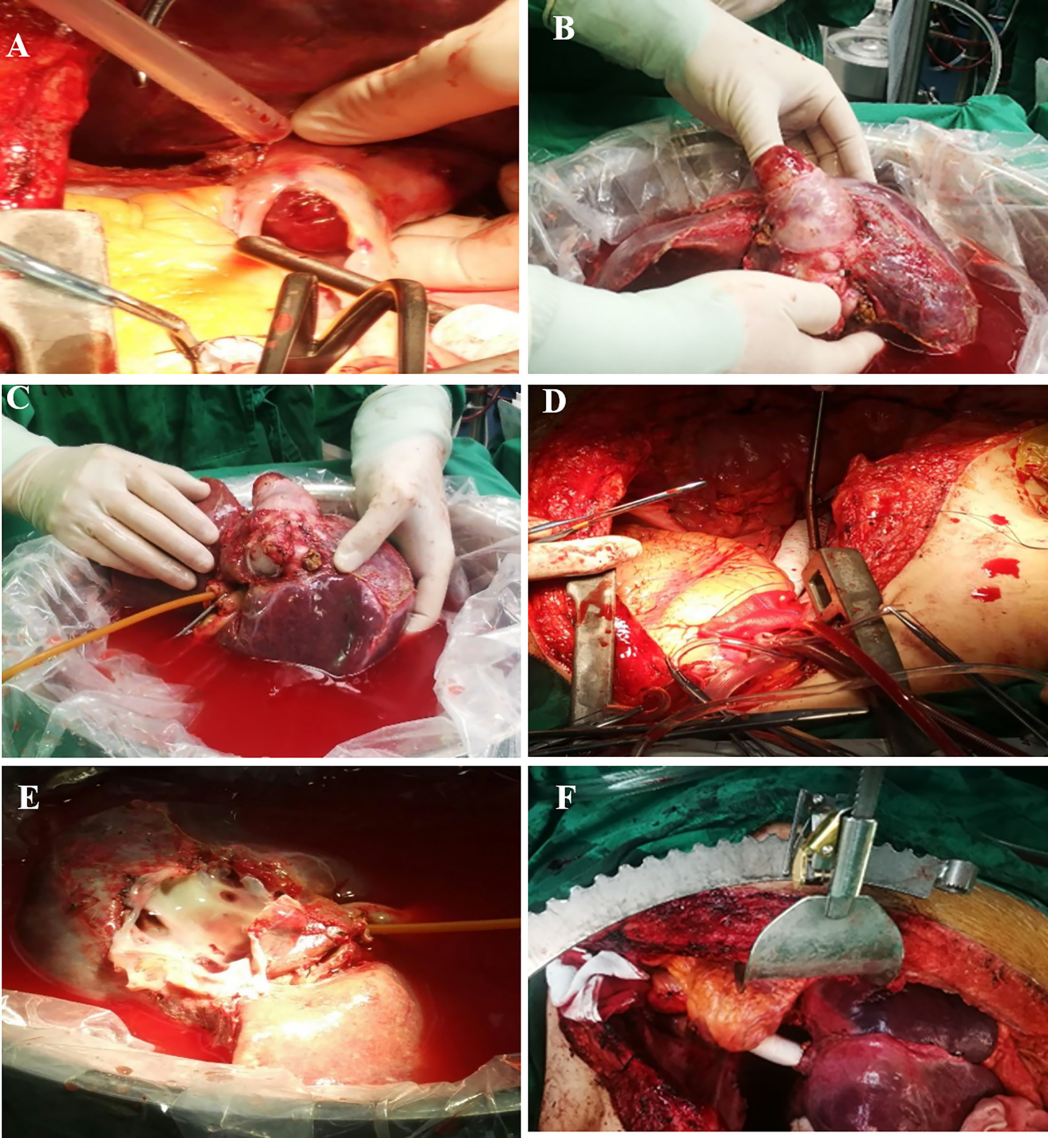
Surgical resection of zone II-III leiomyosarcoma. **(A)**, leiomyosarcoma protruding into right atrium; **(B)**, e*n-blo*c resection of IVC with whole liver; **(C)**, bench resection of tumor and hypothermic perfusion; **(D)**, IVC reconstruction with prosthetic graft; **(E)**, hepatic veins’ orifices after leiomyosarcoma resection; **(F)**, re-implanted liver graft.

After successful anesthesia, right subcostal reverse “L” shaped incision with midline extension was taken. An amount of 2,000-ml ascites was found and cleared after entering the abdomen. A nodular liver without obvious tumor involvement to parenchyma was observed and intraoperative ultrasound was carried out to rule out any potential hepatic lesions. Frozen section pathology for both lobes of liver has shown chronic passive congestion. Then, a median sternotomy was performed and anterior pericardium was opened. The transplant team dissected the liver and *vena cava* circumferentially, freeing the IVC from the retroperitoneum as well as diaphragm. Major vasculatures were carefully dissected, and hepatic artery, portal vein, common bile duct, and intrahepatic *vena cava* were totally freed and suspended. No significant bleeding occurred during the mobilization of the liver. CPB was then initiated after systemic heparinization *via* cannulation of ascending aorta, right atrium, and femoral vein. Clamps were placed on the infra-hepatic IVC below the margin of the lesion but above the bilateral renal vein levels, as well as hepatic artery and portal vein. Then, right atrium was incised above the tumor margin, and a vent was placed in coronary sinus. The hepatic inflow and outflow were disconnected for explanting the whole liver with the tumor (**Figures 2A, B**).

At this time, two independent teams performed bench resection and maintained hemodynamic stability, respectively. While the bench resection was occurring, the atrium was reconstructed and narrowing of the coronary sinus was avoided. Then a Gore-Tax graft was interpositioned between the stump of the right atrium and the infra-hepatic *vena cava*. Then temporary porto-systemic shunting was performed between the portal trunk and prosthetic graft in end-side fashion in order to decompress the portal pressure. Thereafter, the cannulas were removed and CPB was terminated. The total CPB time was 84 min (**Figures 2C–E**).

As soon as the whole liver was procured, it was placed in ice basin and flushed with histidine-tryptophan-ketoglutarate (HTK) solution *via* portal vein and hepatic artery. The thrombi in three hepatic veins were removed by using Fogarty embolectomy catheter and sent to frozen section examination which excluded tumor thrombi (**Video 1**). The tumor was originated from the posterior wall of IVC and found to invade adjacent hepatic tissue with 2 cm. This was managed with regional hepatectomy. Retro-hepatic *vana cava* with leiomyosarcoma was completely resected sparing partial anterior wall of IVC and hepatic veins’ orifices without tumor; resection margin was confirmed by repeated frozen section. On the bench, the remaining wall of IVC was sutured and formed a “funnel shape” IVC, leaving the orifice for hepatic veins as outflow. After the completion of bench resection, the autograft was re-implanted and the orifice was directly anastomosed to prosthetic graft. Temporary porto-systemic shunt was then discontinued, and portal vein, hepatic artery, and common bile duct were anastomosed in end-to-end fashion. By this time, the clamps were removed and autograft was perfused (**Figure 2F**). The anhepatic time was 128 min and total duration of surgery was 12 h and 10 min. The intraoperative blood loss was 1500 mL, resulting in the perfusion of four units of red blood cells, 990 mL fresh frozen plasma (FFP), and 9.25 cryoprecipitate. Mediastinal and abdominal drainage tubes were placed prior the closure of the incision. Postoperative pathology confirmed the diagnosis of leiomyosarcoma. The patient was sent to ICU and extubated at postoperative day 3. Low-molecular heparin was administered as a prophylactic measure to prevent thrombosis from the fourth day. At postoperative day 10, pericardiocentesis was performed due to medium pericardial. Postoperative ultrasound showed patent hepatic veins, portal vein, hepatic artery, and IVC. She was discharged at postoperative day 21 with normal liver and heart function with oral diuretics. At her 3-month postoperative follow-up, she was ambulant daily without shortness of breath. CT scan at postoperative 10 months showed no recurrence and metastasis. Unfortunately, hepatic nodules representing metastasis were discovered on surveillance scans 24 months postoperatively for which the patient underwent percutaneous radiofrequency ablation. She is now 32 months after surgery with regular follow-up.

## Discussion

Primary leiomyosarcomas of IVC originate from the smooth muscle of vascular wall and present as a rare clinical entity (8). They usually grow slowly and asymptomatically and, at the time of diagnosis, become very large in size and extend into renal, hepatic veins, and atrium resulting in compromised liver and renal function, making curative surgical resection difficult (9). However, radical resection is the only curative option with longer survival considering the limited responsiveness to cytotoxic chemotherapy and radiotherapy (1, 10).

Historically, most of the leiomyosarcomas of IVC, especially within zone II and/III, were considered inoperable due to extensive involvement of the liver and right atrium and a multi-visceral *en bloc* resection with complicated vascular reconstruction are required. Although postoperative recurrence is common, a pooled data analysis of 377 surgically resected leiomyosarcomas of IVC claimed that radical resection is the best treatment modality and, in many circumstances, can present a complex procedure requiring a multi-disciplinary team to manage appropriately (11). First reported by Pichlmayr in 1988 (5), ELRA is now mainly considered for tumors with extensive involvement to hepato-caval region that require complicated reconstruction. In a previous study, we have reported single center experience with 69 patients undergoing ELRA with 100% R0 resection rate and 7.2% 30-day mortality rate (12). Similarly, Zawistowski et al. (13) reported data in 244 patients undergoing ELRA with 98.6% R0 resection rate and 7.9% 30-day mortality.

ELRA was first introduced to the management of zone II-III leiomyosarcoma aiming to achieve R0 resection and longer survival (14). On review of the literature, seven relevant case reports could be identified (**Table 1**). Leiomyosarcoma with cephalad extension to hepato-caval region, hepatic veins, and right atrium are the main reasons for ELRA (19). Veno-venous bypass was commonly reported and CPB considered as long as atrial reconstruction was needed and/or IVC calming was impossible due to extreme extension to pericardial IVC (17, 19). Multi-organ resection and combined bilateral kidney autotransplantation were reported (18, 20) as an extreme radical procedure with acceptable survival. Despite the frequent use of veno-venous bypass in these reported cases, it seems not to be necessary. Studies showed temporary IVC reconstruction and porto-systemic shunt may provide better hemodynamic control and zero bypass related complications (6, 12). No mortality was reported and all patients were alive (6-53) months after ELRA despite of recurrence (**Table 2**). Liver and renal dysfunctions are common complications after ELRA. Our case experienced postoperative liver dysfunction mainly due to patient's compromised preoperative liver function prior surgery. No postoperative liver dysfunction was reported in literature; however, renal dysfunction was reported after unilateral *en bloc* resection (20). Preoperative liver dysfunction is regarded as one of the relative contraindications to ELRA. However, surgical resection was preceded due to the short-term manifestation for Budd-Chiari syndrome, and normal previous background of patients. Besides, the liver dysfunction secondary to hepatic vein occlusion due to thrombi and leiomyosarcoma could be reversed as long as successful vascular reconstruction is performed.

**Table 1:** Summary of reported cases on the baseline information of leiomyosarcomas.

Publication	Age	Gender	Symptoms	Location	Size	Previous surgery	Reason for ELRA
Brekke, I. B (14)	64	Male	Leg edema	Zone II–III	6 cm × 6 cm × 7 cm	No	Extension to hepato-caval region
Gruttadauria, S (15)	58	Male	Back pain	Zone II–III	3 cm × 4 cm × 5 cm	IVC resection; Right hepatectomy	Extension to hepatic veins and supre-hepatic *vena cava*
Cho, S (16)	55	Male	Back pain, leg edema	Zone II–III	7.5 cm	IVC ligation	Extension to supre-hepatic *vena cava* and liver metastasis
Takatsuki, M (17)	40	Female	Leg edema	Zone I–III	NG	No	Three hepatic veins circumference by the tumor
Fernandez, H. T (18)	52	Female	Leg edema, dyspnea	Zone I–III	15 cm	No	Three hepatic vein involvement and portal vein invasion
Bunting, B (19)	53	Female	Abdominal distention, leg edema	Zone II–III	8.8 cm × 4 cm × 5 cm	No	The intrahepatic vena cava with right atrium extension
Buchholz, B. M (20)	58	Female	Abdominal mass	Zone I–II	5.7 cm × 5.7 cm × 11 cm	No	Extension to hepatic veins and renal veins
Present study	33	Female	Abdominal distention, leg edema	Zone II–III	10 cm × 6.5 cm × 4.5 cm	No	Extension to hepato-caval region, three hepatic veins and right atrium

**Table 2:** Summary of reported cases on operative parameters and clinical outcomes of leiomyosarcomas.

Publication	Need for extracorporeal circulation	Vascular substitute	Organ perfusion	Anhepatic time	Operative time	Blood loss	Synchronous surgery	Hospital stay	Morbidity	Recurrence	Chemotherapy	Re-surgery	Current status	Follow-up
Brekk, I. B. (14)	F-P-A	Prosthetic graft	UW	283	458	NG	No	11	No	No	No	No	Alive	24
Gruttadauria, S (15)	P-J	No	HTK	145	540	NG	Hepatectomy	10	No	No	No	No	Alive	NG
Cho, S (16)	NG	NG	HTK	NG	NG	NG	No	NG	No	Y	Yes	Lung lobectomy Hepatic RFA	Alive	53
Takatsuki, M (17)	F-P-A	Prosthetic graft	HTK	180	840	15500	No	NG	No	No	No	No	Alive	6
Fernandez, H. T (18)	F-P-A	Prosthetic graft, cryopreserved aorta and iliac vein	HTK	NG	NG	NG	Bilateral renal autotransplantation	NG	No	No	Yes	No	Alive	12
Bunting, B (19)	CPB	Pericardium graft	HTK	166	813	6000	Atrial reconstruction	10	No	Yes	Yes	No	Alive	14
Buchholz, B. M (20)	No	Cryopreserved IVC graft;	HTK	120	575	NG	Right renal resection	NG	Renal dysfunction	Yes	No	No	Alive	24
Our study	CPB	Prosthetic graft	HTK	128	730	1500	Hepatectomy+atrial reconstruction.	21	Liver dysfunction	Yes	No	Hepatic RFA	Alive	32

Recurrence after surgical resection occurs in nearly all patients, with a 5-year disease free survival (DFS) of only 6%. However, long-term survival was possible after radical resection, with 5-year overall survival (OS) of 55% (11). In literature, two patients received ELRA after recurrence of leiomyosarcoma after preliminary surgical management and a patient surviving 6 years after initial surgery and 4 years after ELRA. Liver and lung metastasis are the most commonly reported and re-resection and radiofrequency ablation (RFA) seemed to be a better option (15, 16).

Long-term survival is possible after ELRA for IVC leiomyosarcoma. Hepatic veins thrombi and Budd-Chiari syndrome secondary to leiomyosarcoma does not necessarily preclude the option for ELRA. Recurrence of this rare tumor is common, and resection should be considered in light of the tumor biology and the general condition of the individual patient.

## Data Availability Statement

The original contributions presented in the study are included in the article/**Supplementary Material**. Further inquiries can be directed to the corresponding authors.

## Ethics Statement

The studies involving human participants were reviewed and approved by Ethical committee of First Affiliated Hospital of Xinjiang Medical University. The patients/participants provided their written informed consent to participate in this study.

## Author Contributions

TT and TL drafted the manuscript. SA, Y-BH, LB, and S-SG performed literature search. Z-PW, TT, and TL analyzed the data. TT, TL, QH, JW, and J-MZ performed the surgery. J-MZ, TL and TT critically revised the manuscript. All authors contributed to the article and approved the submitted version.

## Funding

This work was supported by grants from Xinjiang Uyghur Autonomous Region Key Laboratory Open Research Program (2018D04002); Tian Shan Youth Program (2017Q094); Shu-Lan Excellent Project-Support Program for Overseas Study of Young Talents in Organ Transplantation, 2017; and Xinjiang Natural Science Funding (2021D01C299).

## Conflict of Interest

The authors declare that the research was conducted in the absence of any commercial or financial relationships that could be construed as a potential conflict of interest.

## Abbreviations

3D, three dimensional; ALT, alanine aminotransferase; AST, aspartate aminotransferase; CA, carbohydrate antigen; CT, computed tomography; CPB, cardiopulmonary bypass; ELRA, *ex vivo* liver resection and autotransplantation; HV, hepatic veins; IVC, inferior vena cava; PT, prothrombin time; PTA, prothrombin time activity; RA, right atrium.
